# Prophage φEr670 and Genomic Island GI_Er147 as Carriers of Resistance Genes in *Erysipelothrix rhusiopathiae* Strains

**DOI:** 10.3390/ijms27010250

**Published:** 2025-12-25

**Authors:** Marta Dec, Aldert L. Zomer, Marian J. Broekhuizen-Stins, Renata Urban-Chmiel

**Affiliations:** 1 Department of Veterinary Prevention and Avian Diseases, Faculty of Veterinary Medicine, University of Life Sciences in Lublin, Głęboka 30, 20-612 Lublin, Poland; renata.urban@up.edu.pl; 2 Division of Infectious Diseases and Immunology, Utrecht University, 3584 CG Utrecht, The Netherlands; a.l.zomer@uu.nl (A.L.Z.);; 3 WHO Collaborating Centre for Reference and Research on Campylobacter and Antimicrobial Resistance from a One Health Perspective/WOAH Reference Laboratory for Campylobacteriosis, 3584 CL Utrecht, The Netherlands

**Keywords:** resistance genes, genomic island, ICEs, prophage, antimicrobial susceptibility, *Erysipelothrix*

## Abstract

In this study we employed nanopore whole genome sequencing to analyze the resistance genes, genomic islands and prophage DNA in two multidrug resistant *E. rhusiopathiae* strains, i.e., 670 and 147, isolated from domestic geese. MLST profiles and core-genome phylogeny were determined to assess strain relatedness. In strain 670 (serotype 8, ST 113), a novel 53 kb prophage φEr670 carrying the *lnuB* and *lsaE* resistance genes was identified. Regions highly homologous to the φEr670 prophage were detected in 36 of 586 (6.14%) publicly available *E. rhusiopathiae* genomes, as well as in some other Gram-positive bacteria, and usually contained resistance genes. *E. rhusiopathiae* strain 147 (serotype 5, ST 243) was found to contain a composite 98 kb genomic island (GI_Er147) carrying the *ant(6)-Ia* and *spw* genes, as well as gene encoding a putative lincosamide nucleotidyltransferase designated *lnu(J)* and a *vat* family gene encoding a putative streptogramin A O-acetyltransferase. The *lnu(J)* gene exhibited 83.6% homology to the *lnu(D)* gene, and *lnu(J)*-positive *E. rhusiopathiae* strains displayed intermediate susceptibility to lincomycin. *Vat*-positive strain 147 and *vat*-negative *E. rhusiopathiae* strains showed similar susceptibility to quinupristin/dalfopristin. The presence of the Tn916 transposon carrying the *tetM* gene was confirmed in the genomes of both *E. rhusiopathiae* strains; in strain 147, however, Tn916 was located within ICEEr1012. Based on analyses of additional *E. rhusiopathiae* genomes, the integration sites of Tn916, ICEEr1012, and GI_Er147 were identified as genomic “hot spots,” contributing to the genome plasticity of *E. rhusiopathiae*. Prophage φEr670 and GI_Er147 as well as the Tn916 transposon and ICEEr1012 are most likely responsible for the dissemination of resistance genes in *E. rhusiopathiae*. Prophages highly homologous to φEr670 act as carriers of resistance genes in various Gram-positive bacteria. However, the transferability of the identified genetic elements and the functional role of the *lnu(J)* gene require further investigation. This study provides new insights into the diversity of MGEs in *E. rhusiopathiae* and advances understanding of the genomic mechanisms driving antimicrobial resistance in Gram-positive bacteria.

## 1. Introduction

*Erysipelothrix rhusiopathiae* is a Gram-positive bacterium responsible for erysipelas in a wide range of hosts, including poultry, swine, and other domestic and wild animals, and it also poses a zoonotic risk to humans [1]. In poultry, infections with *E. rhusiopathiae* can cause acute septicaemia, sudden mortality, and chronic disease manifestations, leading to significant economic losses in waterfowl and turkey production [2,3,4]. Outbreaks of erysipelas are typically controlled using antimicrobial therapy, with penicillins remaining the treatment of choice [2,5]. However, the increasing detection of antimicrobial resistance (AMR) among *E. rhusiopathiae* isolates has raised growing concerns regarding the effectiveness of current therapeutic strategies, with potential implications for both animal health and public health [5]. Moreover, the lack of commercially available vaccines specifically approved for the prevention of erysipelas in geese and ducks [5] reinforces the dependence on antimicrobial therapy and emphasizes the need for continuous surveillance of AMR in this pathogen. Accordingly, a deeper understanding of the molecular mechanisms driving AMR and horizontal gene transfer in *E. rhusiopathiae* is crucial.

The acquisition and dissemination of resistance determinants in bacteria are often mediated by mobile genetic elements (MGEs), such as prophages, plasmids, genomic islands, and integrative and conjugative elements (ICEs), including transposons. These elements not only provide a reservoir of AMR determinants but also facilitate their horizontal transfer across strains and even species boundaries [6]. Prophages, in particular, are increasingly recognized as important vehicles of resistance genes in Gram-positive bacteria [7,8], while ICEs represent versatile elements capable of integrating into bacterial chromosomes and mediating conjugative transfer. To date, several MGEs carrying AMR genes have been identified in *E. rhusiopathiae*, including the bacteriophage φ1605, ICEEr0106 [9], ICEEr1023 and ICEEr1012 [10]. However, the diversity and functional roles of MGEs in this species, and their contribution to shaping its *resistome*, remain far from fully understood.

In our previous work, we characterized the antimicrobial susceptibility and resistance gene content of *E. rhusiopathiae* isolates from waterfowl, identifying multidrug-resistant (MDR) strains carrying both resistance genes and phage- or ICE-associated sequences. In serotype 8 isolate 670, the *lsaE* gene was located adjacent to phage-related genes, whereas serotype 5 isolate 147 harbored conjugation-related genes together with resistance determinants such as *ant(6)-Ia* and *spw* [5]. Encouraged by these observations, we carried out the present study to perform detailed genomic analyses of these *E. rhusiopathiae* isolates (670 and 147). We identified and characterized a novel prophage (φEr670) and a novel composite genomic island (GI_Er147), both associated with resistance genes, as well as the well-known transposon Tn916 carrying *tetM*. Furthermore, we examined the distribution of φEr670-like prophages in publicly available genomes of *E. rhusiopathiae* and related Gram-positive bacteria. Collectively, these analyses provide new insights into the contribution of MGEs to resistance gene carriage and dissemination in *E. rhusiopathiae*, and highlight their broader potential role in the spread of AMR among Gram-positive bacteria.

## 2. Results

### 2.1. Basic Genomic Analyses, ST Determination and Detection of Resistance Genes

The genome size of *E. rhusiopathiae* strains 670 serotype 8 and 147 serotype 5 was 1,874,230 bp and 1,918,116 bp, respectively, and the GC content was 36.3–36.6%. Both genomes contained 55 tRNAs with 3–7 rRNA loci (Table 1). These results are consistent with reports by other authors that the genome size of *E. rhusiopathiae* strains ranges from 1.6 to 1.9 Mb and the GC content ranges from 36% to 38%; the number of tRNAs ranges from 53 to 57 and the number of rRNA operons from 3 to 9 [10,11,12], NCBI database, The Ribosomal RNA Database (https://rrndb.umms.med.umich.edu, accessed on 15 July 2025).

ST 113 strains such as strain 670 were previously found to express serotype 8 and were isolated from waterfowl and pigs in Poland [5,13]. Globally, this sequence type appears to be rare: among 557 *E. rhusiopathiae* isolates analyzed, ST113 has been identified only in three serotype 1b strains from Canada [5]. ST 243 identified in strain 147 with serotype 5 was previously detected in only one of 557 *E. rhusiopathiae* strains, i.e., strain 267 with serotype 5 isolated from geese in Poland [10].

Genotypic resistance profiles obtained from WGS data were consistent with the results of previous PCR-based detection of resistance genes [5]. The multidrug-resistant strain 670 confirmed the presence of *tetM*, *lnuB*, and *lsaE* genes, while strain 147 contained *tetM*, *ant(6)-Ia*, and *spw* genes (Table 1).

### 2.2. Detection of Prophage Regions

Using the PHASTER tool, three regions containing incomplete prophage DNA were detected in the genome of *E. rhusiopathiae* strain 147 (PHASTER score < 60), while in strain 670 two prophage regions were detected (PHASTER score 80 and 150). One was highly homologous to the *Erysipelothrix* phage SE-1 (GB: NC_029078.1; size 33,997 bp) (score 150—intact), the second –designated φEr670—showed some similarity to the *Corynebacterium* Adelaide phage (GB: NC_048791.1; 44 kb) (score 80—questionable). The sequence of both prophages (SE-1 and φEr670) contained a clearly higher percentage of GC than (>40%) than the whole genome DNA (36.6%), indicating horizontal gene transfer.

**Figure 1:**
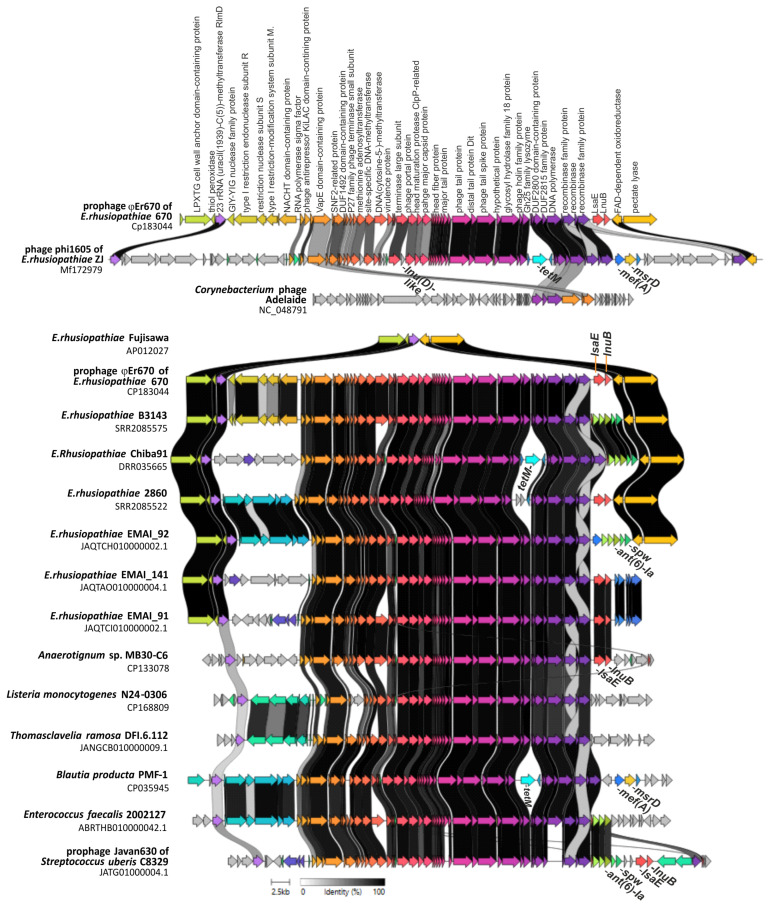
Clinker visualization of homology between prophage φEr670 of *E. rhusiopathiae* strain 670 and phage φ1605 and *Corynebacterium* phage Adelaide as well as φEr670-like prophages of selected *E. rhusiopathiae* strains and strains of other Gram-positive bacteria. Arrows represent genes; the arrow’s colors represent the gene clusters identified by Clinker; homology between genes is represented by a gray gradient (%, the scale at bottom of the figure).

The size of the φEr670 prophage was estimated at 53,020 bp (GB: CP183044.1:544708-597727), and further analyses using BLAST (https://blast.ncbi.nlm.nih.gov/Blast.cgi, accessed on 4 June 2025) showed its significant similarity to the sequence of phage Javan630 (48 kb) infecting *Streptococcus uberis* [14] and to phage φ1605 (90,000 bp, GB: MF172979.1) infecting *E. rhusiopathie* [9] (Figure 1). Both of these phages represent the class *Caudoviricetes* [9,14]. 

**Figure A1:**
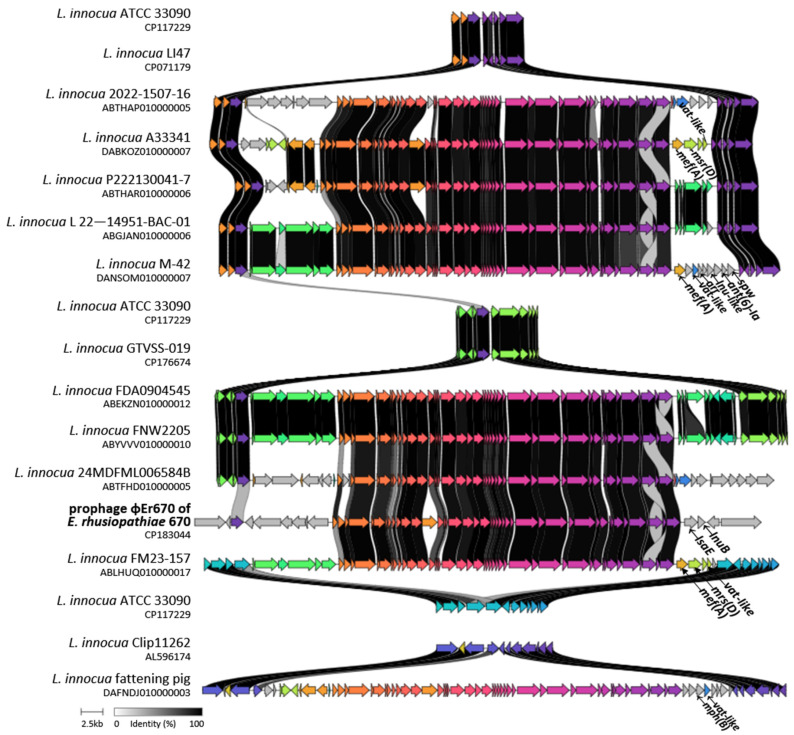
Clinker visualization of prophage regions in *Listeria innocua* strains and their homology with the prophage φEr670 of *E. rhusiopathiae* 670. Arrows represent genes; the arrow’s colors represent the gene clusters identified by Clinker; homology between genes is represented by a gray gradient (%, the scale at the bottom of the figure).

Regions highly homologous (≥89.9%) to the φEr670 prophage were detected in the genomes of 36 *E. rhusiopathiae* strains out of 586 (6.14%) examined, and in 1 out of 30 strains representing other *Erysipelothrix* species (Table S1). The φEr670-like prophages were also found in two strains of *Thomasclavelia ramosa* as well as in some genomes of Gram-positive bacteria outside the *Erysipelothrixales* family, i.e., *Listeria*, *Anaerotignum*, *Eubacterium*, *Enterococcus* and *Blautia* (Figure 1, Table 2). A notably high prevalence of φEr670-like prophages was observed in *Listeria innocua*; BLAST searches identified prophage-positive regions in at least 100 publicly available genomes of this species (Figure A1). 

The size of φEr670-like prophage regions detected in *E. rhusiopathiae* strains (*n* = 36/586) and other Gram-positive bacteria ranged from 50 to 59 kb (excluding the larger 90 kb φ1605 phage) (Table 2), which is consistent with their classification as medium-sized dsDNA phages [15].

Within the φEr670 prophage, two AMR genes, i.e., *lsaE* and *lnuB*, were identified. Similarly, in most of the 36 *E. rhusiopathiae* strains carrying φEr670-like prophages, prophage-associated DNA also contained resistance determinants, including *lsaE* and *lnuB* (*n* = 12/36). In 17 genomes the prophage contained the *ant(6)-Ia* and *spw* genes, while in 11 genomes the *tetM* or *mefA* gene was additionally present. The *tetM*, *mefA*, *msrD* and *lnuD*-like genes were present in the φ1605 prophage of *E. rhusiopathiae* ZJ, and strain EMAI_31 had two homologous prophage regions, one containing the *msrD* and *ermG* resistance genes and the other the *mphB* gene (Table 2 and Table S1). Various resistance genes (*lnuB*, *lsaE*, *ant6-Ia*, *spw mefA*, *msrD*, *mphB*, *tetM*, *arr*, and putative resistance genes *vat*-like and *lnu*-like) were present within φEr670-like prophages detected in the genomes of Gram-positive bacteria outside the *Erysipelothrix* genus (Table 2, Figure 1).

**Figure 2:**
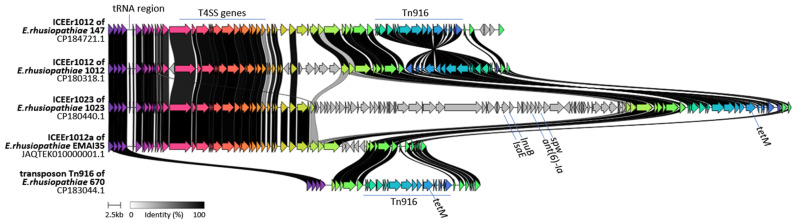
Clinker visualization of the location of the *tetM*-carrying transposon Tn916 in *E. rhusiopathiae* strains 147 (within ICEEr1012) and strain 670. Comparison of Tn916-containing regions to homologous MGEs found in other *E. rhusiopathiae* strains and integrated into the same chromosomal “hot spot”. Arrows represent genes; the arrow’s colors represent the gene clusters identified by Clinker; homology between genes is represented by a gray gradient (%, the scale at bottom of the figure).

Comparative genomic analysis revealed a high degree of structural conservation across φEr670 and its homologous prophages identified in *E. rhusiopathiae* and other Gram-positive bacteria. As shown in Figure 2, extensive synteny blocks span the major functional modules, including those involved in DNA replication, genome packaging (terminase subunits), virion assembly (capsid and tail proteins), and host–cell lysis. They correspond to the 38–46 core genes (out of 51 identified in φEr670) that were consistently present across the 36 prophage–positive *E. rhusiopathiae* genomes (Table S1). In contrast, genomic variation is largely confined to the accessory regions near the prophage termini, which exhibit substantial differences in gene content. Such divergence likely reflects intra-lineage modular exchange, including the acquisition of AMR genes, and is consistent with the natural genetic variability commonly observed among closely related prophages [15,16]. BLAST-based comparisons demonstrated 63–93% query coverage and 89.9–95.7% nucleotide identity relative to φEr670 (Table 2), and the corresponding orthoANI values ranged from 87.8 to 97.2% (or 92.7–97.2% for *E. rhusiopathiae* prophages when the divergent φ1605 phage is excluded). Together, these metrics indicate that the φEr670-like prophages form a cohesive evolutionary group, with most elements falling within or just below the ~95% ANI threshold often used to delineate species in dsDNA phage taxonomy [16].

**Table 2:** List of bacterial strains containing DNA regions homologous to the φEr670 prophage and the size of the prophages, its similarity to the φEr670 prophage and associated resistance genes.

Taxonomic Group	Strain	Prophage Size [kb]	Resistance Genes Located Within Prophage Regions	Homology with Phage φEr670		Ortho ANI Value [%]	GenBank Acc. No.	Reference
				Query Cover [%]	Similarity [%]			
*E.rhusiopathiae* (prophageφEr670)	670	53	*lsaE*, *lnuB*	NA	NA	100	CP183044.1	This study
*E. rhusiopathiae* (prophage φ1605)	ZJ	90	*mef(A)*, *msr(D)*, *lnu(D)-like*, *tetM*	63	92.7	87.8	MF172979.1	[9]
*E. rhusiopathiae*	EMAI_29	51	*mph(B)*	71	94.2	94.9	JARGDV010000003.1	[17]
*E. rhusiopathiae*	EMAI_31	~52	*msr(D)*, *erm(G)*	76	95.2	ND	JAQTEO010000002.1	[17]
*E. rhusiopathiae*	EMAI_31	~52	*mph(B)*	70	95.3	94.4	JAQTEO010000011.1	[17]
*E. rhusiopathiae*	EMAI_33	51	*mph(B)*	70	95.3	94.4	JAQTEM010000002.1	[17]
*E. rhusiopathiae*	EMAI_91	53	*lsaE*, *lnuB*	82	94.4	94.5	JAQTCI010000002.1	[17]
*E. rhusiopathiae*	EMAI_92	54	*spw*, *ant(6)-Ia*, *mef(A)*	72	94.9	95.1	JAQTCH010000002.1	[17]
*E. rhusiopathiae*	EMAI_141	53.5	*lsaE*, *lnuB*	78	94.7	95.1	JAQTAO010000004.1	[17]
*E.rhusiopathiae*	B3129	54.5	*spw*, *ant(6)-Ia*	75	95.0	95.1	SRR2085573	UN
*E.rhusiopathiae*	B3142	54.5	*spw*, *ant(6)-Ia*	75	95.0	95.1	SRR2085574	UN
*E.rhusiopathiae*	B5577	54	*spw*, *ant(6)-Ia*	75	95.3	95.1	SRR2085578	UN
*E.rhusiopathiae*	B3143	54	*spw*, *ant(6)-Ia*	93	95.0	95.3	SRR2085575	UN
*E.rhusiopathiae*	B3144	50	none	72	92.1	92.7	SRR2085576	UN
*E.rhusiopathiae*	B3159	51	*lsaE*, *lnuB*	81	91.4	91.5	SRR2085577	UN
*E.rhusiopathiae*	G2	52.5	*lsaE*, *lnuB*	89	94.6	94.7	SRR2085593	UN
*E.rhusiopathiae*	2604	51	*lsaE*, *lnuB*	78	94.7	94.7	SRR2085518	UN
*E.rhusiopathiae*	2628	52	*lsaE*, *lnuB*	82	95.2	95.4	SRR2085520	UN
*E.rhusiopathiae*	2860	53	*lsaE*, *lnuB*	78	94.3	94.4	SRR2085522	UN
*E.rhusiopathiae*	6028	53	*lsaE*, *lnuB*	78	94.6	94.5	SRR2085524	UN
*E.rhusiopathiae*	6106	52	*lsaE*, *lnuB*	80	91.9	91.8	SRR2085525	UN
*E.rhusiopathiae*	Chiba 91	59	*spw*, *ant(6)-Ia*, *tetM*	73	92.7	92.9	DRR035665	[18]
*E.rhusiopathiae*	Saitama 91	59	*spw*, *ant(6)-Ia*, *tetM*	73	92.7	92.9	DRR035666	[18]
*E.rhusiopathiae*	Chiba 92A	59	*spw*, *ant(6)-Ia*, *tetM*	73	92.7	92.9	DRR035667	[18]
*E.rhusiopathiae*	Chiba 92B	56.5	*spw*, *ant(6)-Ia*, *tetM*	73	94.1	94.7	DRR035668	[18]
*E.rhusiopathiae*	Chiba 93	59	*spw*, *ant(6)-Ia*, *tetM*	73	92.7	ND	DRR035669	[18]
*E.rhusiopathiae*	Kanagawa 95	58	*spw*, *ant(6)-Ia*, *tetM*	73	92.7	ND	DRR035671	[18]
*E.rhusiopathiae*	Nagano 98	59	*spw*, *ant(6)-Ia*, *tetM*	73	92.7	94	DRR035672	[18]
*E.rhusiopathiae*	Saitama 01	59	*spw*, *ant(6)-Ia*, *tetM*	73	92.7	ND	DRR035675	[18]
*E.rhusiopathiae*	16BKT031005	55	*ermG*, *msr(D)*, *tetM*	72	94.0	ND	ERR3932976	[19]
*E.rhusiopathiae*	16BKT031013	51.5	*lnuB*, *lsaE*	82	95.7	95.3	ERR3932981	[19]
*E.rhusiopathiae*	16BKT31009	54	*lnuB*, *lsaE*	77	94.8	ND	ERR3932985	[19]
*E.rhusiopathiae*	17MIK0642341	52	none	73	94.6	94.7	ERR3932998	[19]
*E.rhusiopathiae*	17MIK0642351	54.5	*spw*, *ant(6)-Ia*	75	95.0	ND	ERR3932999	[19]
*E.rhusiopathiae*	17MIK0642361	51.5	*lnuB*, *lsaE*	82	95.2	95.4	ERR3933000	[19]
*E.rhusiopathiae*	17MIK0642371	54.5	*spw*, *ant(6)-Ia*	75	95.0	ND	ERR3933001	[19]
*E.rhusiopathiae*	swine100	52	*spw*, *ant(6)-Ia*	77	96.1	97.2	ERR3678831	[20]
*E.rhusiopathiae*	swine29	55	*spw*, *ant(6)-Ia*, *mefA*	72	93.4	ND	ERR3678845	[20]
*Erysipelothrix larvae*	LV19	~59	none	76	94.4	ND	CP013213.1	[21]
*Thomasclavelia ramosa*	DFI.6.112	53	none	72	94.9	94.2	JANGCB010000009.1	UN
*Thomasclavelia ramosa*	DFI.6.30	53	none	72	94.9	94.2	JAJCKK010000013.1	UN
*Streptococcus uberis* (prophage Javan630)	C8329	52	*lsaE*, *lnuB*, *spw*, *ant(6)-Ia*	77	95.5	94.8	JATG01000004.1	UN
*Anaerotignum* sp.	MB30-C6	ND	*lsaE*, *lnuB*	81	95.6	94.6	CP133078.1	[22]
*Eubacterium callanderi*	DSM 2594	ND	none	74	94.6	ND	CP132136.1	[23]
*Eubacterium callanderi*	DSM 2593	ND	none	74	94.6	ND	CP132135.1	[23]
*Eubacterium limosum*	EI1405	57	none	75	93.7	94.2	CP171347.1	UN
*Eubacterium limosum*	DFI.6.107	54	none	72	93.9	94.1	JAJCLO010000002.1	UN
*Listeria monocytogenes*	N24-0306	56	none	69	89.9	88.3	CP168809.1	[24]
*Blautia producta*	PMF-1	ND	*mef(A)*	82	95.2	ND	CP035945.1	UN
*Clostridiaceae* bacterium	HFYG-1003	ND	*mef(A)*, *msr(D)*	71	94.6	ND	CP102060.1	UN
*Enterococcus faecalis*	2002127	58	*ant(6)-Ia*	74	92.6	90.1	ABRTHB010000042.1	UN
*Listeria innocua*	2022-1507-16	54	none	72	94.8	ND	ABTHAP010000005.1	UN
*Listeria innocua*	A33341	54	*mef(A)*, *msr(D)*, *vat*-like	76	95.6	94.8	DABKOZ010000007.1	[25]
*Listeria innocua*	24MDFML00 6584B	ND	none	72	93.6	ND	ABTFHD010000005.1	UN
*Listeria innocua*	fattening pig	54	*mph(B), vat*-like, *lnu(J)*-like	75	95.3	ND	DAFNDJ010000003.1	[25]
*Listeria innocua*	FDA1205947- C002-006	56	none	71	89.9	ND	ABJLEO010000012.1	UN
*Listeria innocua*	FDA0904545	55.5	none	71	92.9	92.9	ABEKZN010000012.1	UN
*Listeria innocua*	FM23-157	53	*mef(A)*, *msr(D)*, *vat*-like	71	95.5	ND	ABLHUQ010000017.1	UN
*Listeria innocua*	FNW2205	55	none	68	92.9	93	ABYVVV010000010.1	UN
*Listeria innocua*	22-014951-BAC-01	53	none	71	95.6	94.7	ABGJAN010000006.1	UN
*Listeria innocua*	P222130041-7	51	none	78	94.7	94.3	ABTHAR010000006.1	UN
*Listeria innocua*	M-42	55.5	*mef(A)*, *vat*-like, *arr*, *ant(6)-Ia*, *spw*, *lnu*-like	68	91.8	92.2	DANSOM010000007.1	[25]

NA—not applicable; ND—not determined; ANI—average nucleotide identity; UN—unpublished.

The φEr670 prophage and φEr670-like prophages were characterized by a specific integration site (*att*). In *E. rhusiopathiae* strain 670 and other *E. rhusiopathiae* strains (except strains EMI_91 and EMAI_141), the prophage DNA was flanked by a gene encoding 23S rRNA (uracil(1939-(C5)) methyltransferase RlmD [its product catalyzes the formation of 5-methyl-uridine at position 1939 (m5U1939) in 23S rRNA] and a gene encoding FAD-dependent oxidoreductase. The RlmD methyltransferase gene also flanked prophage DNA in the genomes of other Gram-positive bacteria, indicating a key role of this gene in prophage DNA integration. (Figure 1 and Figure A1).

In our previous study, we confirmed the presence of three genes of prophage φEr670 (encoding the major capsid protein, a minor tail protein, and a site-specific recombinase) as well as the adjacent region encompassing the phage recombinase and *lsaE* gene in 7 out of 60 *E. rhusiopathiae* isolates recovered from waterfowl and in 1 out of 14 isolates from pigs. Notably, these three prophage genes were found exclusively in multidrug-resistant strains (*lsaE-lnuB-tetM*) of serotype 8 and ST113 [5]. In contrast, isolates from geographically distant regions that carried φEr670-like prophages represented other serotypes, most often 1a (10/36), 1b (11/36) and 2 (11/36), (Table S1). These findings suggest that the infection of *E. rhusiopathiae* strains with φEr670 phage is not dependent on serotype-specific receptor recognition. Instead, the occurrence of φEr670-positive strains of serotype 8 and ST113 on several poultry and swine farms in Poland may reflect clonal expansion and passive maintenance of integrated prophage DNA, rather than recent active transduction events.

The results of our analyses suggest that prophages of the φEr670 lineage play an important evolutionary role in shaping the resistome of *E. rhusiopathiae* and may contribute more broadly to the dissemination of antimicrobial resistance across Gram-positive bacteria. The phenomenon of AMR gene transfer via bacteriophage-mediated transduction is increasingly well documented and understood [26,27]. In *Erysipelothrix rhusiopathiae*, this mechanism was demonstrated by Gu et al. [9], who showed mitomycin C–induced transfer of the φ1605 prophage carrying the *mef(A)*, *msr(D)*, and *tetM* resistance genes from strain ZJ to strain G4T10. Similarly, future studies may assess whether the φEr670 prophage is capable of producing progeny phages that could infect *E. rhusiopathiae* strains and mediate the transfer of the *lnuB* and *lsaE* genes. To date, the occurrence of these resistance determinants within prophage-associated DNA has been reported only in *Streptococcus pyogenes* [28]. The present study therefore provides the first evidence of *lnuB* and *lsaE* being located within prophage sequences in *E. rhusiopathiae* as well as in *Streptococcus uberis* and *Anaerotignum* sp. (Table 1).

The detection of prophages of φEr670 family across distinct Gram-positive bacterial species is an intriguing finding that warrants further investigation. Bacteriophages have traditionally been considered narrow-host-range viruses, often restricted to specific strains of single species [29]. However, increasing evidence points to the existence of broad host range phages that contribute to interspecies gene transfer. For example, *Listeria* phage A511 targets a wide range of *Listeria* species [30] while *Staphylococcus* phage K infects multiple coagulase-positive and coagulase-negative staphylococci [31]. Similarly, wastewater-derived staphylococcal phages were shown to infect multiple *Staphylococcus* species [32], and phage Φm46.1 is able to transfer between *S. suis* and *S. pyogenes* [33]. Mechanistically, such broadened host tropism may result from the utilization of conserved surface receptors (e.g., wall teichoic acids), structural adaptability of tail fiber or baseplate proteins, and the deployment of anti-restriction or anti-CRISPR mechanisms that allow phages to circumvent taxon-specific bacterial defense systems [34,35]. Together, these observations—combined with the presence of φEr670-like prophages not only in *E. rhusiopathiae* but also across multiple Gram-positive bacterial species—strongly support the hypothesis that this phage lineage is capable of cross-species transfer. Such interspecies mobility positions φEr670-related prophages as important evolutionary drivers shaping the resistome of *E. rhusiopathiae* and potentially contributing to the broader dissemination of AMR among Gram-positive bacteria.

### 2.3. Detection of ICEs and Genomic Islands

In *Erysipelothrix rhusiopathiae* strains 670 and 147, an 18 kb *tetM*-carrying Tn916 transposon was identified. It showed >99.7% sequence similarity to Tn916 elements described in other Gram-positive bacteria, including *E. rhusiopathiae* [9,10,36,37,38]. In strain 670, the Tn916 transposon occurred as an independent element, whereas in strain 147, it was located within the 80 kb ICEEr1012 element, previously identified in *E. rhusiopathiae* strain 1012 isolated from geese in Poland [10]. The ICE_Er1012 element containing Tn916 was additionally detected in *E. rhusiopathiae* strain EMAI_31, while a variant lacking Tn916 (designated ICEEr1012a) was found in several *E. rhusiopathiae* isolates of swine origin from Australia (EMAI_35, 159, 130, 131, 133, 134, 135, 136, 171, 172, and 180) [17] (Figure 2). The presence of ICEEr1012a and Tn916 in association with a resistance gene cluster was also previously confirmed on the large (130 kb) genomic island ICEEr1023, identified previously in *E. rhusiopathiae* strain 1023 [10]. All of these MGEs (Tn916, ICEEr1012a, ICEEr1012, and ICEEr1023) were integrated at a specific chromosomal site located in the vicinity of tRNA-encoding genes (Figure 2), suggesting that this region may represent a genomic “hot spot” for the acquisition and recombination of MGEs in *E. rhusiopathiae*.

**Figure 3:**
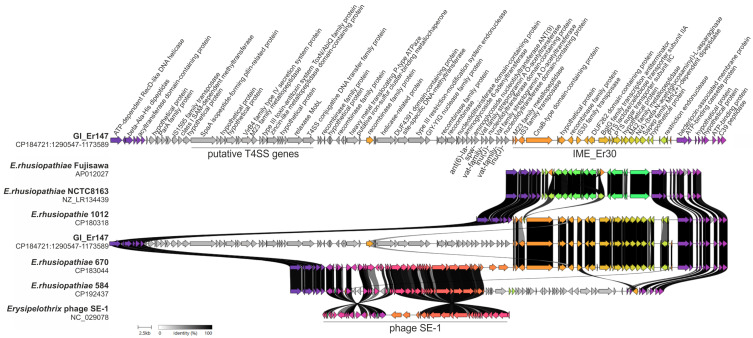
Genomic island GI_Er147, containing IME_Er30, the T4SS gene region, and the resistance gene cluster, compared with homologous MGEs present in other *E. rhusiopathiae* strains and integrated at the same chromosomal “hot spot”. Arrows represent genes; the arrow’s colors represent the gene clusters identified by Clinker; homology between genes is represented by a gray gradient (%, the scale at bottom of the figure).

A 98 kb genomic island was detected in *E. rhusiopathiae* strain 147 (CP184721.1: 1185333-1283798) and designed as GI_Er147. It carries resistant genes, i.e., *ant(6)-Ia*, *spw*, *vat*-like gene and *lnu*-like gene as well as genes involved in bacterial conjugation, i.e., genes encoding recombinases, transposases, relaxase MobL and the genes found on type IV secretion systems (T4SS) (Figure 2). However, it is not known whether this is the complete set of genes enabling the formation of protein machinery for DNA transfer between bacterial cells. Therefore, this element was classified as a genomic island and not as an ICE (Figure 3).

**Figure A2:**
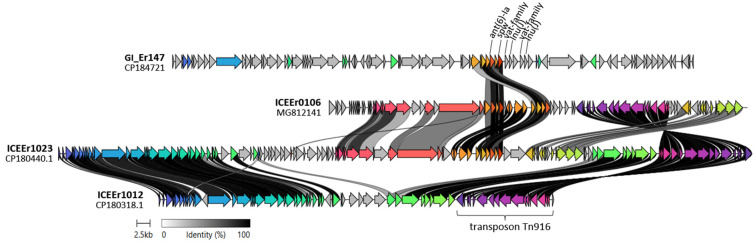
Homology between GI_Er147 and ICEs previously detected in *E. rhusiopathiae* strains. Arrows represent genes; the arrow’s colors represent the gene clusters identified by Clinker; homology between genes is represented by a gray gradient (%, the scale at the bottom of the figure).

GI_Er147 showed only little similarity to the genomic islands previously found in *E. rhusiopathiae*, i.e., ICEEr1023, ICEEr1012 [10] and ICEEr0106 [9] (Figure A2).

Within GI_Er147, a 30 kb integrative mobile element was identified, harboring two transposase genes (belonging to the IS3 and IS30 families) and a recombinase-family gene, which together suggest a potential capacity for mobility. This element, designated IME_Er30, was detected also in strain 670 and 3 other *E. rhusiopathiae* strains from geese in Poland (1023, and 267), in several dozen Australian isolates [17], and in *E. amsterdamensis* strain A18Y020d (GB: OW659496.1). In contrast, IME_Er30 was not found in the reference strains *E. rhusiopathiae* Fujisawa and NCTC 8163 (ATCC 19414), nor in isolates originating from China (ZJ, WH13013, ML101, SY1027, GAT10, SE38, and GXBY-1). The functions of IME_Er30 remain unknown, but this element may contribute to the acquisition of additional adaptive traits in *E. rhusiopathiae*. In the genomes of *E. rhusiopathiae* strains 670, EMAI_5, and EMAI_51, IME_Er30 is located adjacent to prophage SE-31, forming a hybrid IME–prophage element (Figure 3). In *E. amsterdamensis* strain A18Y020d, as well as in *E. rhusiopathiae* strain 147, IME_Er30 is positioned next to a large integrative element carrying Type IV Secretion System (T4SS) genes. IME_Er30, the SE-1 prophage, and hybrid elements formed through the combination of IME_Er30 with other MGEs such as GI_Er147, were all integrated at the same chromosomal locus in *E. rhusiopathiae*, located near the gene encoding tRNA 2-thiouridine(34) synthase MnmA. This integration site, similar to the insertion site of the Tn916 transposon and larger Tn916-containing ICEs, may represent a genomic “hot spot” for horizontal gene transfer in *E. rhusiopathiae* (Figure 3). Similar preferential integration near tRNA or tRNA-related genes has been widely reported for both prophages and integrative elements, as these loci contain short, conserved sequence motifs (*att* sites) that are efficiently recognized by integrases [39,40]. The coexistence of prophages and IMEs at the same chromosomal position also supports the concept of mosaic or hybrid genomic islands, which arise through sequential integration and recombination events between different MGEs [41].

Within GI_Er147, the presence of several resistance genes was demonstrated, i.e., *ant(6)-Ia* (*aadE*, coding for aminoglycoside nucleotidyltransferase ANT(6)-Ia), *spw* (encoding for ANT(9) spectinomycin adenyltransferase), *vat* family gene encoding putative streptogramin A O-acetyltransferase (2 copies), and gene encoding nucelotidyltransferase domain-containing protein (2 copies). The coexistence of *ant(6)-Ia* and *spw* genes noted in these studies is common in Gram-positive bacteria [10]. In *E. rhusiopathiae* they can be located on large genomic islands (e.g., ICEEr1023 and Er0106) [9,10,37], including prophages of φEr670 family [this study] (Figure 1 and Figure A2). 

**Figure 4:**
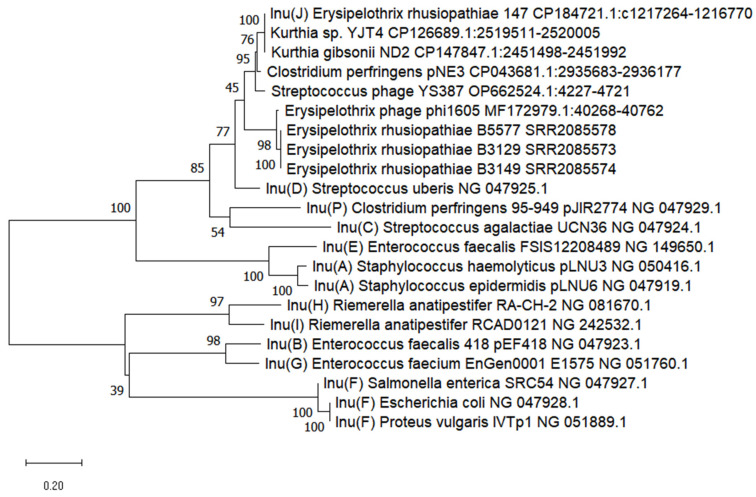
Dendrogram showing the similarity of the *lnu(J)* gene sequence of *E. rhusiopathiae* 147 to reference genes from the *lnu* family derived from the GenBank database (with the prefix NG in the acc. No.) and *lnu*-like genes found in other bacterial strains. The percentage of replicate trees in which the associated taxa were clustered together in the bootstrap test (200 replicates) is shown next to the branches. Scale bars show genetic distance.

The gene encoding nucelotidyltransferase domain-containing protein (GB: CP184721.1 locus_tag AC-PCEN_09575 and ACPCEN_09590), designed as *lnu(J)* (495 bp, GB: PV882487), showed similarity to the *lnu* family genes encoding lincosamide nucleotidyltransferase, with the highest homology, i.e., 83.6% (100% coverage), with the *lnu(D)* gene (GB: NG_047925.1, 513 bp) (Figure 4). Genes homologous to *lnu(J)* gene (83–100% homology) were found in strains of various Gram-positive bacteria, i.e., *Kurthia*, *Streptococcus*, *Anaerocolumna*, *Clostridium*, and in phages infecting streptococci, as well as in several *E. rhusiopathiae* strains, i.e., ZJ (within prophage φ1605), and B3129, B3142 and B5577 (Figure 4). Unfortunately, susceptibility of these strains to lincosamides is unknown.

PCR analysis of 60 wild-type isolates of *E. rhusiopathiae* from waterfowl [5], including strains 147 and 670, showed that the *lnu(J)* gene was present only in two strains representing serotype 5, i.e., strain 147 and 136. Both strains were characterized by intermediate resistance to lincomycin, i.e., MIC 4–8 µg/mL and were susceptible to clindamycin (Table 3) [5].

The MIC for lincomycin of the *E. rhusiopathiae* strains in which neither the *lnu(J)* nor the *lnu(B)* gene was detected (*n* = 49/61, including 60 wild-type strains and reference strain *E. rhusiopathiae* ATCC 19414) was in the range 0.25–1 µg/mL, while the *lnu(B)*-positive *E. rhusiopathiae* strains (*n* = 9/61) are characterized by very high lincomycin MIC values, i.e., >64 µg/mL (with a simultaneous clindamycin MIC of 1–4 µg/mL) (Table 3) [5]. Despite these indications of the involvement of the *lnu(J)* gene in the reduction in the susceptibility of *E. rhusiopathiae* strains to lincomycin, the unambiguous definition of the role of the *lnu(J)* gene requires additional analyses.

It was previously shown that the *lnu(D)* gene, to which the *lnu(J)* gene shows 83.6% homology, was responsible for the moderate sensitivity of *S. uberis* UCN 42 strain to lincomycin (MIC = 2 µg/mL) (MIC for *lnuD*-negative *S. uberis* 72 strain was 0.06 µg/mL). Introduction of the recombinant pUC18 plasmid with the cloned *lnu(D)* gene into *E. coli* resulted in a change in the lincomycin MIC from 32 µg/mL to 128 µg/mL [42].

**Figure A3:**
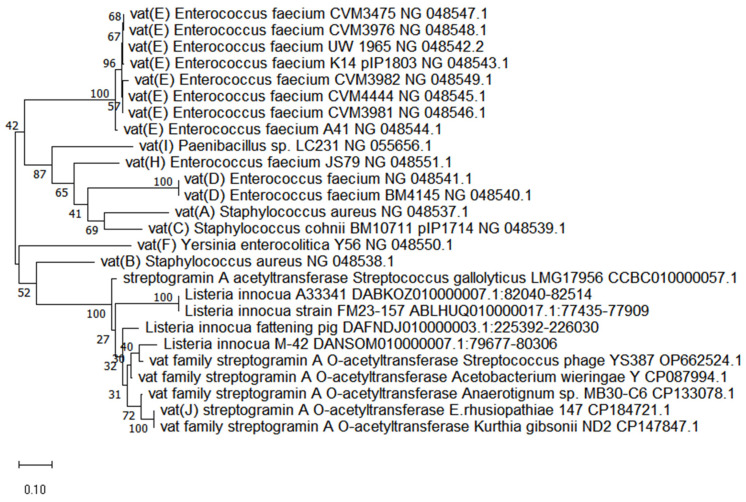
A dendrogram showing the similarity of the *vat*-like gene (encoding a putative streptogramin A O-acetyltransferase) from GI_Er147 to reference *vat* family genes retrieved from the GenBank database (accession numbers prefixed with “NG”) and to vat-like genes identified in other bacteria. The percentage of replicate trees in which the associated taxa were clustered together in the bootstrap test (200 replicates) is shown next to the branches. Scale bars show genetic distance.

The gene encoding putative streptogramin A O-acetyltransferase, located within GI_Er147 showed 71.2% (89% coverage) similarity to the *vat(B)* gene (GB: NG_048538.1) and lower homology to other genes of the *vat* family (Figure A3). This gene was also detected in the genomes of other Gram-positive bacteria (deposited in the GenBank database), and in some strains (*Kurthia* and *Bacillus*, and within the phage YS387 detected in *Streptococcus suis* where it was located next to the *lnu(J)* gene. However, our studies did not confirm that the *vat* family gene of GI_Er147 confers resistance to streptogramins. Phenotypic testing using E-tests showed no difference in the sensitivity of strain 147 and *vat*-negative strains to quinupristin/dalfopristin (mixed streptogramins type A and B) (Table 3). It is also possible that the gene could also not be expressed as its promoter is not well recognized or because it encodes a different functionality as it is only 71.2% similar to known *vat* genes.

**Figure A4:**
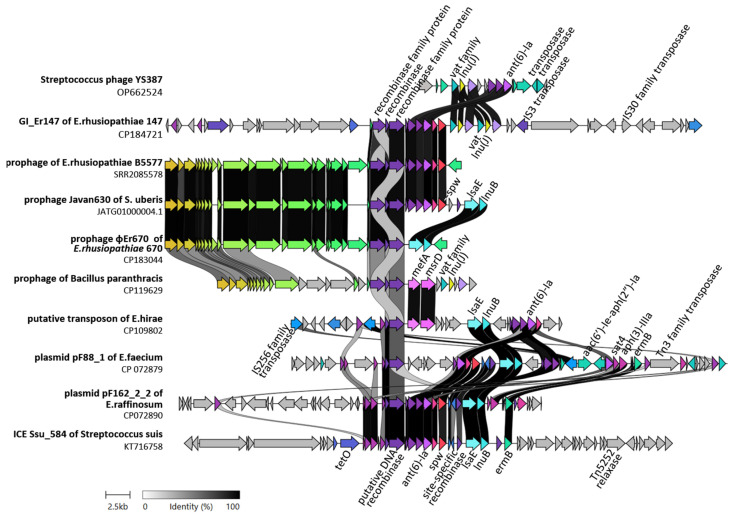
Clinker visualization of homology between recombinases located within GI_Er147, prophage φEr670, and other MGEs found in various Gram-positive bacteria. Arrows represent genes; the arrow’s colors represent the gene clusters identified by Clinker; homology between genes is represented by a gray gradient (%, the scale at the bottom of the figure).

It is worth emphasizing that the serine recombinase encoding gene within GI_Er147 shows high homology to the recombinase of prophage φEr670 and related phages in other *Erysipelothrix* strains, as well as to recombinases carried by diverse MGEs (phages, plasmids, transposons and other ICEs) found in other Gram-positive bacteria (Figure A4). This pattern reflects the strong evolutionary conservation of serine recombinases, which mediate site-specific integration and excision. Unlike tyrosine recombinases, which use a stepwise cleavage–religation mechanism, serine recombinases catalyze coordinated double-strand breaks and strand exchange in a single concerted reaction [43], making them particularly efficient modules for horizontal movement across MGEs.

### 2.4. Core Genome Phylogeny

**Figure 5:**
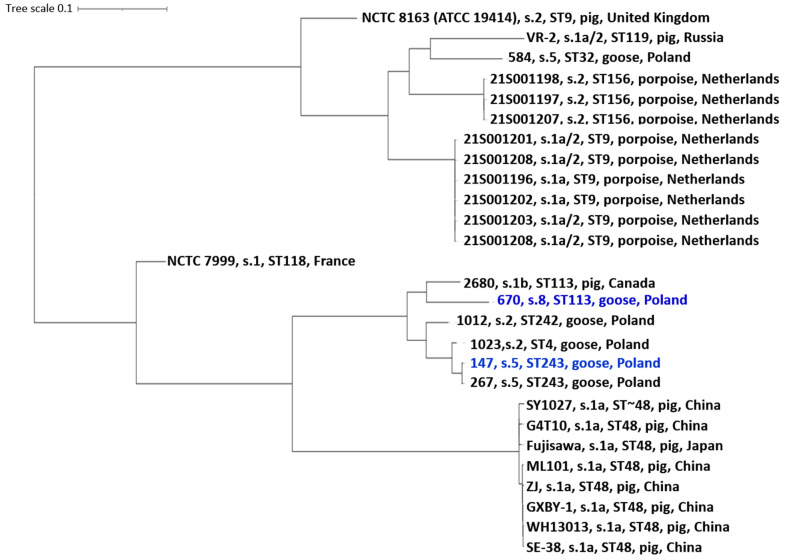
Phylogenetic tree of a 1,528,858 bp whole genome alignment of *E. rhusiopathiae* strains 670 and 147 (marked in blue), compared with 25 genomes of *E. rhusiopathiae* strains whose sequences were obtained from the GenBank database (the accession numbers are listed in Table S1). s.—serotype; ST—sequence type.

The phylogenetic tree based on the core-genome alignment clearly separates *E. rhusiopathiae* isolates into several distinct clades corresponding to their sequence types (STs) and geographic origins. All Polish isolates, except for strain 584 (ST32), form a single, well-supported branch, indicating a close genomic relationship. Strains 147 (ST243) and 670 (ST113) show the greatest similarity to isolates from Poland and Canada sharing the same STs. This clustering pattern suggests a common evolutionary background of Polish goose isolates, distinct from pig-derived strains from China and Japan and from porpoise isolates originating from the Netherlands. The reference strains NCTC 8163 (serotype 2, ST9, United Kingdom) and NCTC 7999 (serotype 1, ST118, France) form separate branches, while isolates from Asia (e.g., Fujisawa, ZJ, ML101) group together within the ST48 lineage (Figure 5).

## 3. Materials and Methods

### 3.1. Isolation, Identification, and Phenotypic Characterization of E. rhusiopathiae Strains

The two isolates of *E. rhusiopathiae* included in this study, i.e., 670 serotype 8 and 147 serotype 5, were isolated in 2020 from internal organs of domestic geese that were delivered dead to the laboratory for diagnostic purposes [2].

Strains *E. rhusiopathiae* 670 and 147 were selected from a pool of 60 *E. rhusiopathiae* isolates from waterfowl based on phenotypic and genotypic resistance profiles as well as detection of putative phage genes (coding for major capsid protein and minor tail protein) and ICEs related genes (*mobL*, *int-Tn*). The serotype of the strains was previously determined using multiplex PCR [5].

### 3.2. Whole Genome Sequencing

Nanopore sequencing was performed according to protocol SQK-RBK110.96 with Flow Cell ver. R10 on a MinION device (FLO-MIN106D; Oxford Nanopore, Oxford, UK), using the super-accurate base-calling method in MinKNOW v22.12.7. Reads were trimmed and down-sampled to 200× coverage using Filtlong (https://github.com/rrwick/Filtlong, accessed on 12 May 2025) and assembled into circular contigs using Flye v2.9.1 [44]. Genomes were polished using Medaka (https://github.com/nanoporetech/medaka, accessed on 4 June 2025) and Homopolish [45] and annotated using NCBI Prokaryotic Genome Annotation Pipeline (PGAP) [46]. The genome assemblies of both strains were deposited in the GenBank database (NCBI) under accession numbers CP183044.1 (strain 670) and CP184721.1 (strain 147).

### 3.3. Detection of Resistance Genes, Genomic Islands and Prophage DNA

Resistance genes were previously identified using PCR in *E. rhusiopathiae* strains 147 and 670 [5]. In this work, more detailed detection of resistance genes was performed based on WGS analysis using Resfinder 4.1 [47] and Resistance Gene Identifier (RGI) ver. 6.0.3 [48]. Synonymous mutations in the *gyrA* gene were determined by aligning amino acid sequences predicted using ORF Finder (https://www.ncbi.nlm.nih.gov/orffinder/, accessed on 12 June 2025). Genomic islands of *E. rhusiopathiae* strains 670 and 147 were identified by aligning the whole genome sequences of these strains with those of the reference strains *E. rhusiopathiae* Fujisawa and NCTC 8163 using the BLAST tool (https://blast.ncbi.nlm.nih.gov/Blast.cgi, accessed on 1 October 2025). Prophage DNA was detected in the genomes of the *E. rhusiopathiae* strain 147 and 670 using the PHAge Search Tool—Enhanced Release (PHASTER) [49]. Regions homologous to the φEr670 prophage were detected in the genomes of *E. rhusiopathiae* strains (*n* = 586) and other *Erysipelothrix* species (*n* = 30) retrieved from the GenBank database (Table S1) using Kaptive v3 and a custom database containing the annotated sequence of φEr670 [50]. 

### 3.4. Determination of Susceptibility to Streptogramins and Lincosamides

The susceptibility of strain 147 (*vat*-family positive) and several other *E. rhusiopathiae* strains with known genomic sequence to streptogramins was determined using E-tests containing quinupristin/dalfopristin (Liofilchem, Roseto degli Abruzzi, Italy). The test was performed on blood agar with a bacterial inoculum of 0.5 McFarland density. The plates were incubated for 24 h at 37 °C, 5% CO_2_. The susceptibility of the bacteria to lincomycin and clindamycin was determined using the broth microdilution method [5]. The antimicrobial susceptibility test was performed in duplicate.

### 3.5. Detection of lnu(J) Gene

The *lnu(J)* gene identified in the genome of strain 147 was detected by PCR in 60 wild-type *E. rhusiopathiae* isolates listed in our previous paper [5]. The negative control was the reference strain *E. rhusiopathiae* ATCC 19414 (NCTC 8163), whose genome is deposited in the GenBank database (NZ_LR134439.1). Amplification of *lnu(J)* gene was performed using the following primers: forward 5’-TTGGATAGATGGCGGTTGGG-3’ and reverse 5’-ACTTGTATTCACTCGGAACAGGAA-3’. The PCR product size was 431 bp. For 5 wild type isolates whose WGSs are publicly available (nos. 1023, 1012, 584, 267, 670), detection was based also on in silico analysis. 

### 3.6. Determination of Homology Between DNA Sequences

The similarity of the MGEs detected in strains 670 and 147 to the sequences found in the genomes of other *E. rhusiopathiae* strains as well as other bacterial species was determined using BLAST (https://blast.ncbi.nlm.nih.gov/Blast.cgi, accessed on 13 September 2025) and Clinker [51].

Average nucleotide identity (ANI) between prophage φEr670 and homologous prophages was calculated with OrthoANI [52] using online calculator (www.ezbiocloud.net/tools/ani, accessed on 6 December 2025).

The phylogenetic relationships between the *lnu(J)* and *vat*-family genes identified in *E. rhusiopathiae* strain 147 and reference *lnu* and *vat* genes were inferred using the Maximum Likelihood method implemented in MEGA ver. 11 [53].

### 3.7. Multilocus Sequence Typing 

Multilocus sequence typing (MLST) of *E. rhusiopathiae* strains was performed according to the scheme developed by Webster et al. [17] using mlst v.2.19.0 (https://github.com/tseemann/mlst, accessed on 18 June 2025).

### 3.8. Phylogenetic Inference

The phylogenetic analysis included the *E. rhusiopathiae* strain 670 and 147 from geese and 25 other *E. rhusiopathiae strains*, including several MDR strains which, like strain 670, contained the *lsaE*, *lnuB* and *tetM* genes. Two of these strains, i.e., ZJ and 2860, contained regions homologous to the φEr670 prophage, and strain 2860, like strain 670, represented ST113. In turn, strain 267 represents the same sequence type as strain 147, i.e., 243. WGSs of 25 *E. rhusiopathiae* strains used for comparative analysis were downloaded from the GenBank database (accession numbers in Table S1). Whole genome alignments were performed using parsnp v2.0 [54]. Phylogenetic trees were constructed using Fasttree v 2.1.11 [55] and visualized using iTOL v5 [56].

## 4. Conclusions

In this study, we provide a comprehensive whole-genome characterization of MGEs associated with AMR in two *E. rhusiopathiae* strains, i.e., multidrug-resistant serotype 8 strain 670 and the serotype 5 strain 147, isolated from domestic geese. We report the identification of a previously undescribed prophage, φEr670 (53 kb), carrying the *lnuB* and *lsaE* resistance genes, as well as a novel hybrid genomic island, GI_Er147 (98 kb), harboring several resistance determinants and T4SS genes. Together with the detection of Tn916 (18 kb), ICEEr1012 (80 kb), and two chromosomal “hotspots” that promote the integration and recombination of diverse MGEs, these findings reveal a previously underappreciated complexity of the *E. rhusiopathiae* mobilome.

The widespread distribution of φEr670-like prophages—most of which also carry AMR genes—strongly suggests that this phage lineage plays an important role in shaping the resistome of *E. rhusiopathiae* and other Gram-positive bacteria.

Overall, this study provides new insights into the diversity and organization of MGEs in *E. rhusiopathiae*, thereby advancing current understanding of the genomic mechanisms underlying the evolution and dissemination of AMR in this species and potentially across Gram-positive bacteria. Further studies are warranted to elucidate the mobility of the newly identified genetic elements and the functional significance of the *lnu(J)* gene encoding a putative lincosamide nucleotidyltransferase.

## Figures and Tables

**Table 1:** Basic information about the WGSs of strains 147 and 670 and the results of the MLST analysis, antimicrobial susceptibility test and detection of resistance genes and MGEs.

Isolate ID (Genome ID)	670 (24S01951-1)	147 (24S1949-1)
**GenBank Acc. No.**	CP183044.1	CP184721.1
**Serotype**	8	5
**Source/host**	Domestic goose	Domestic goose
**Year of isolation, country**	2020, Poland	2020, Poland
**Genome size (bp)**	1,874,230 bp	1,918,116 bp
**Genes (total)**	1842	1871
**Genes (coding)**	1753	1795
**tRNAs**	55	55
**rRNAs**	7, 7, 7 (5S, 16S, 23S)	3, 3, 3 (5S, 16S, 23S)
**GC content (%)**	36.6	36.3
ST (MLST)	113	243
**Antimicrobial susceptibility test results (MIC in μg/mL) ^a^**	**TET(32), LIN(>64), CLI(2), TIA(>64)**, ERY(0.125), **ENR(8)**, AMP(≤0.06) QDA(0.5), STR(128), SPE(64)	**TET(32)**, LIN(8) ^b^, CLI(0.125), TIA(0.5), ERY(0.125), **ENR(8)**, AMP(≤0.06) QDA(0.38), STR(>512), SPE(512)
**Resistance genes**	*tetM*, *lnuB*, *lsaE*	*tetM*, *ant(6)-Ia*, *spw*, *lnu(J)* **^c^**, *vat*-family **^c^**
**Mutations in *gyrA* gene** **& in *parC* gene**	Thr86 → Ile Ser81 → Ile	Thr86 → Lys Ser81 → Ile
**MGEs**	Prophage φEr670 (*lnuB*, *lsaE*); Tn916 (*tetM*); Genomic island containing prophage SE-1 and IME_Er30 (no resistance genes)	GI_Er147 containing T4SS-positive region and IME_Er30 (*ant(6)-Ia*, *spw*, *lnu(J)* **^c^**, *vat*-family **^c^**); ICEEr1012 containing T4SS-positive region and Tn916 (*tetM*)

^a^ numbers in brackets refer to the MIC value expressed in µg/mL; bold values indicate resistance; ^b^ value indicating moderate susceptibility to antibiotics. [5] ^c^ putative resistance genes. AMP—ampicillin, CLI—clindamycin, ENR—enrofloxacin, ERY—erythromycin, LIN—lincomycin, QDA—quinupristin/dalfopristin, SPE—spectinomycin, STR—streptomycin, TET—tetracycline, TIA—tiamulin.

**Table 3:** Results of determining the susceptibility of *E. rhusiopathiae* strains to streptogramins and lincosamides.

Strain	GenBank Acc. No.	Resistance Genes	MIC [µg/mL]		
			Lincomycin R ≥ 16 µg/mL I 4–8 µg/mL	Clindamycin R ≥ 1 µg/mL I 0.5 µg/mL	Quinupristin/Dalfopristin
ATCC 19414	LR134439.1	none	0.25–0.5	0.015–0.03	0.38
147	CP184721.1	*tetM*, *ant(6)-Ia*, *spw, lnu(J)* *, *vat-family* *	8	0.06–0.125	0.38
136	NA	*tetM*, *ant(6)-Ia*, *spw*, *lnu(J)* *	4–8	0.125–0.25	0.5
670	CP183044.1	*tetM*, *lnuB*, *lsaE*	>64	2	0.5
1023	CP180440.1	*tetM*, *lnuB*, *lsaE*, *ant(6)-Ia*, *spw*, *erm47*	>64	2	0.5
584	CP192437.1	none	0.5	0.06	0.38
1012	CP180318.1	*tetM*	0.5–1	0.06–0.125	0.38

R—resistant; I—intermediate; NA—not applicable; *—putative resistance genes.

## Data Availability

The original contributions presented in this study are included in the article/Supplementary Material. Further inquiries can be directed to the corresponding author.

## Supplementary Materials

The following supporting information can be downloaded at: https://www.mdpi.com/article/10.3390/ijms27010250/s1. References [57,58,59,60,61,62,63,64,65,66,67,68,69,70,71] are cited in the Supplementary Materials.
